# Prevalence of postpartum glucose intolerance and its association with physical activity levels among women with prior gestational diabetes mellitus attending public primary care clinics in Penang: A cross-sectional study

**DOI:** 10.51866/oa.880

**Published:** 2025-12-11

**Authors:** Gayatherri Meganathan, Valli Ragunathan, Kaur Ranjit Singh Jaspreet, Balasundram Radhini, Adeline Chen Mei Tai, Khairatul Nainey Kamaruddin, Salim Hani

**Affiliations:** 1 MBBChBAO, MFamMed, PhD ,Department of Family Medicine, Faculty of Medicine and Health Sciences, Universiti Putra Malaysia (UPM), Jalan Universiti 1, Serdang, Selangor, Malaysia. Email: hanisyahida@upm.edu.my; 2 MBBChBAO, MMed Fam Med, Faculty of Medicine, Universiti Teknologi MARA, Jalan Hospital, Sg Buloh, Selangor, Malaysia. Email: khairatul@uitm.edu.my; 3 MBBS, Klinik Kesihatan Kepala Batas, Lot 1466, Mukim 6, Kepala Batas, Penang, Malaysia.; 4 MBBS, Klinik Kesihatan Bandar Tasek Mutiara, Tingkat 1, Bangunan CMART Persiaran Mutiara 4, Bandar Tasek Mutiara, Simpang Ampat, Penang, Malaysia.; 5 MBBS, Klinik Kesihatan Jalan Angsana, Jalan Angsana 3, Ayer Itam, Penang, Malaysia.; 6 MD, Klinik Kesihatan Jalan Macalister Jalan Macalister, Georgetown, Penang, Malaysia.; 7 MBBS, Klinik Kesihatan Kubang Semang, Lot 807, Mukim 5 Kubang Semang, Bukit Mertajam, Penang, Malaysia.

**Keywords:** Postpartum glucose intolerance, Gestational diabetes mellitus, Physical activity

## Abstract

**Introduction:**

Women with a history of gestational diabetes mellitus (GDM) have an increased risk of developing glucose intolerance. This study aimed to assess the prevalence of glucose intolerance and its associated factors among postpartum women with a history of GDM in Penang.

**Methods:**

This cross-sectional study was conducted at five government primary care clinics in Penang. Postpartum women with a history of GDM who underwent a 75-g oral glucose tolerance test (OGTT) at 6-12 weeks postpartum were recruited from August to October 2023. Data collected included sociodemographic details, clinical characteristics, physical activity levels measured using the International Physical Activity Questionnaire-Short Form and OGTT results. Descriptive and multiple logistic regression analyses were performed using IBM SPSS Statistics version 29.

**Results:**

A total of 204 women participated, with a mean age of 31.7 (SD=5.05) years. The prevalence of prediabetes and type 2 diabetes mellitus was 23.5% and 3.9%, respectively. Among the participants, 47.5% were inactive, while 27.5% were physically active. The participants on oral medication or insulin had higher odds of developing postpartum glucose intolerance. Conversely, the participants who were minimally active or active had a lower likelihood of developing glucose intolerance than those who were inactive.

**Conclusion:**

Among women attending primary care clinics in Penang, 27.5% had abnormal glucose tolerance postpartum. Physical inactivity was a significant risk factor. This study highlights the need to promote physical activity during pre-pregnancy care to reduce postpartum glucose intolerance and its complications.

## Introduction

Diabetes is a growing global health concern, with the International Diabetes Federation forecasting an increase from 537 million adults affected in 2021 to 783 million by 2045 - a 46% rise, far exceeding the expected 20% global population growth.^1^ In Malaysia, the prevalence of diabetes surged from 11.2% in 2011 to 18.3% in 2019,^2^ disproportionately affecting women of reproductive age (18-49 years). Among them, gestational diabetes mellitus (GDM) is a leading contributor to hyperglycaemia, accounting for 80% of cases worldwide.^1^ Globally, the prevalence of postpartum glucose intolerance ranges from 20% to 60%, with Malaysian studies reporting rates reaching 12.1%-61.7%, depending on study settings, timing and testing methods.^3,4^

GDM, characterised by glucose intolerance first detected during pregnancy, signals underlying pancreatic P-cell dysfunction, placing women at a tenfold higher risk of developing type 2 diabetes mellitus (T2DM) than those with normoglycaemic pregnancies.^5^ Glucose intolerance includes impaired fasting glucose, (IFG) and impaired glucose tolerance (IGT), and T2DM. Timely screening is essential; thus, women are screened at multiple points. Women with increased risks are screened during booking and again at 24-28 weeks of gestation.^5^ Conversely, women over 25 years old without other risk factors are also screened during this period to manage potential glucose intolerance effectively.^5^ The adverse outcome of GDM is substantial, including preterm delivery, miscarriage, stillbirth, congenital anomalies and preeclampsia, as well as progression to prediabetes and T2DM. Despite these risks, postpartum glucose screening rates remain low, attributed to barriers such as poor compliance, time constraints and limited healthcare access.^6-8^

Several factors significantly contribute to postpartum glucose intolerance, including maternal age, obesity, parity, high insulin needs during pregnancy and sociodemographic variables such as educational attainment and employment status.^4,9,10^ However, no study has yet examined lifestyle factors such as physical activity levels. This study aimed to determine the prevalence and associated factors of postpartum glucose intolerance among women with a history of GDM in Penang. By focusing on this population, the study will enhance the understanding of context-specific factors associated with glucose intolerance, providing a foundation for targeted interventions to reduce diabetes risk in this high-risk group.

## Methods

### Study design and setting

This cross-sectional study involved five government primary health clinics across four districts in Penang that offer maternal and child health services. The study was conducted among postpartum women with a history of GDM who underwent postpartum oral glucose tolerance test (OGTT) from August to October 2023.

### Study population and sampling

Eligible participants were postpartum women with a history of GDM in their last pregnancy who provided consent and could read and write in English or Malay. We excluded women with established diabetes mellitus or overt diabetes, those lost to postpartum follow-up and those with conditions that could affect glucose levels. A convenience sampling method was used in this study. Eligible women scheduled for their routine 6-12-week postpartum OGTT were identified from clinic records. They were approached during their clinic visit, provided study information and invited to participate.

### Sample size calculation

The sample size was calculated using Naing et al.’s formula,^11^ based on the estimated prevalence of abnormal glucose tolerance at 6 weeks postpartum from the study conducted by Fatin and Alina,^6^ resulting in a sample size of 163. For multiple regression analysis, the sample size was calculated using the Tabachnick and Fidell formula,^12^ which yielded a sample size of 154. The higher value of 163 was chosen, and after adjustments for a 20% non-response rate,^13^ the final sample size was 204.

### Research tool and data collection

Self-administered questionnaires in English and Malay languages were distributed to eligible participants. The questionnaires comprised four main components: i) sociodemographic characteristics, ii) clinical characteristics, iii) International Physical Activity Questionnaire-Short Form (IPAQ-SF) scores^14,15^ and iv) postpartum OGTT results.

Sociodemographic data included age, ethnicity, marital status, educational level, economic status and employment status, while clinical data encompassed body mass index (BMI), gestational weight gain, family history of diabetes, obstetric history, parity, GDM treatment and OGTT results at diagnosis.

The IPAQ-SF demonstrates strong test-retest reliability, with intraclass correlation coefficients (ICCs) over 0.80.^16,17^ Compared with objective measures such as accelerometers, the IPAQ-SF shows modest validity, with low correlation coefficients ranging from 0.09 to 0.39. Moderate to strong correlations (0.54-0.92; P<0.001) were noted across intensities and domains, with a K-value of 0.73 for total activity. The PA-Log also exhibited significant validity (P<0.001) across intensities and domains (p=0.67-0.98). These findings confirm the IPAQ-M’s reliability and validity for assessing physical activity in the Malay population.^15^

Participants were assigned research IDs to ensure confidentiality, and all identifiable data were removed. Completed questionnaires were placed in a labelled box at the registration counter, where a research team member reviewed them. Postpartum OGTT results were categorised as normal or abnormal. Abnormal glucose tolerance was defined as a fasting plasma glucose level of ≥6.1 mmol/L and/or a 2-h postprandial glucose level of ≥7.8 mmol/L.

### Data analysis

The prevalence of postpartum glucose intolerance among women with a history of GDM was presented as numbers and percentages. Continuous variables were expressed as means and standard deviations and categorical variables as frequencies and percentages.

A bivariate analysis was conducted to evaluate the factors associated with glucose intolerance in postpartum women with a history of GDM. Continuous variables were analysed using an independent t-test, while categorical variables were assessed using the chi-square test. All variables with a P-value of <0.25 were included in the downstream multivariable analysis.

A multiple logistic regression model was utilised to explore the relationships between the dependent and independent variables. The final regression model included only the factors that remained significant after adjustment. The results were reported as odds ratios with 95% confidence intervals (CIs). A P-value of <0.05 was considered statistically significant. All analyses were performed using IBM SPSS Statistics for Windows, Version 29.0 (IBM Corp., Armonk, NY, USA).

## Results

### Sociodemographic and clinical characteristics

A total of 204 postpartum women with a history of GDM were included in the study. Their sociodemographic and clinical characteristics are summarised in Table 1. The mean age of the participants was 31.7 (SD=5.05) years, and Malays comprised the majority of the population at 76.5%. Half of the participants (50%) had completed their secondary education, and 66.7% were unemployed. Approximately 57.4% belonged to the M40 economic group, while 31.4% were in the B40 group.

**Table 1:** Sociodemographic and clinical characteristics of the participants (N=204).

Sociodemographic variable	n (%)
Age (year), mean (SD)	31.7 (5.05)
**Race**	
Malay	156 (76.5)
Chinese	36 (17.6)
Indian	12 (5.9)
**Marital status**	
Married	204 (100)
Not married (Single, widower, divorcee)	-
**Economic status**	
B40	64 (31.4)
M40	117 (57.4)
T20	23 (11.2)
**Employment status**	
Employed	136 (66.7)
Unemployed	68 (33.3)
**Educational level**	
Primary education	8 (3.9)
Secondary education	102 (50.0)
Tertiary education	94 (46.1)

Clinical and lifestyle variables (during recent pregnancy)	n (%)
**Diagnostic OGTT result,** mean (SD)	
Fasting blood glucose level (mmol/L)	5.1 (0.64)
2-h postprandial glucose level (mmol/L)	8.3 (1.19)
**Parity**	
<5	180 (88.2)
≥5	24 (11.8)
**Body Mass Index (BMI) at booking**	
Underweight	6 (2.9)
Normal	69 (33.8)
Overweight	64 (31.4)
Obesity class 1	47 (23.0)
Obesity class 2	12 (5.9)
Obesity class 3	6 (2.9)
**Gestational weight gain**	
Low	51 (25.0)
Normal	103 (50.5)
Excessive	50 (24.5)
**GDM treatment**	
Diet control	138 (67.6)
Oral medication	54 (26.5)
Combination of oral medication and insulin	12 (5.9)
**Polyhydramnios**	
Yes	12 (5.9)
No	192 (94.1)
**Macrosomic baby**	
Yes	13 (6.4)
No	191 (93.6)
**Congenital anomaly**	
Yes	3 (1.5)
No	201 (98.5)
**Shoulder dystocia**	
Yes	-
No	204 (100.0)
**Intrauterine death**	
Yes	-
No	204 (100.0)
**Usage of corticosteroid during pregnancy**	
Yes	-
No	204 (100.0)
**First-degree family history of DM**	
Yes	151 (74.0)
No	53 (26.0)
**Physical activity**	
Inactive	97 (47.5)
Minimally active	51 (25.0)
Active	56 (27.5)

SD: standard deviation; OGTT: oral glucose tolerance test

In terms of the clinical characteristics, 33.8% of the participants had a normal BMI at the time of their initial antenatal booking; 31.4% were categorised as overweight; and 31.8% had some degree of obesity. Regarding gestational weight gain, 50.5% of the participants maintained normal weight gain during pregnancy, while 24.5% experienced excessive weight gain. Among the treatment modalities for GDM, 67.6% managed their condition through dietary control; 26.5% required oral medication; and 5.9% used a combination of oral medication and insulin.

Additionally, 74.0% of the participants had a first-degree family history of diabetes mellitus, and nearly half (47.5%) were categorised as physically inactive. Key outcomes during the recent pregnancy showed a low prevalence of macrosomic babies (6.4%) and polyhydramnios (5.9%), with no recorded cases of shoulder dystocia or intrauterine death.

### Prevalence of postpartum glucose intolerance

The prevalence of postpartum glucose intolerance among the participants was 27.5% (n=56) (Table 2). Among them, 23.5% had prediabetes, while 3.9% had diabetes.

**Table 2:** Prevalence of glucose intolerance among the participants (N=204).

Postpartum OGTT result	n (%)
Abnormal	56 (27.5)
Prediabetes	48 (23.6)
Diabetes	8 (3.9)
Normal	148 (72.5)

### Factors associated with glucose intolerance

Table 3 presents the results of the bivariate analysis identifying the factors significantly associated with postpartum glucose intolerance. Higher age, higher BMI at booking and excessive gestational weight gain were all significantly linked to increased odds of developing glucose intolerance postpartum. Furthermore, the presence of polyhydramnios and macrosomic babies during pregnancy, along with higher 2-h postprandial glucose levels, demonstrated significant associations with glucose intolerance. Conversely, engaging in physical activity, particularly being moderately or highly active, was associated with a reduced risk of developing glucose intolerance.

**Table 3:** Bivariate analysis of the factors associated with glucose intolerance among the participants (N=204).

Factor	Postpartum OGTT result		Statistical test	P-value
	Abnormal	Normal		
Age (year)	33.2 (5.56)	31.2 (4.74)	2.62^a^	0.009
Race			0.962^b^	0.712
Malay	45 (28.8)	111 (71.2)		
Chinese	9 (25.0)	27 (75.0)		
Indian	2 (16.7)	10 (83.3)		
Economic status			4.11^c^	0.128
B40	21 (32.8)	43 (67.2)		
M40	26 (22.2)	91 (77.8)		
T20	9 (39.1)	14 (60.9)		
Employment status			3.56^c^	0.059
Employed	43 (31.6)	93 (68.4)		
Unemployed	13 (19.1)	55 (80.9)		
Educational level			0.949^b^	0.760
Primary education	1 (12.5)	7(87.5)		
Secondary education	29 (28.4)	73 (71.6)		
Tertiary education	26 (27.7)	68 (72.3)		
Parity			2.76^c^	0.097
<5	46 (25.6)	134 (74.4)		
≥5	10 (41.7)	14 (58.3)		
BMI at booking			17.5^b^	0.005
Underweight	1 (16.7)	5 (83.3)		
Normal	14 (20.3)	55 (79.7)		
Overweight	14 (21.9)	50 (78.1)		
Obesity class 1	15 (31.9)	32 (68.1)		
Obesity class 2	8 (66.7)	4(33.3)		
Obesity class 3	4 (66.7)	2 (33.3)		
Gestational weight gain			16.9^c^	<0.001
Low	10 (19.6)	41 (80.4)		
Normal	21 (20.4)	82 (79.6)		
Excessive	25 (50.0)	25 (50.0)		
GDM treatment			42.1^b^	<0.001
Diet control	20 (14.5)	118 (85.5)		
Oral medication	26 (48.1)	28 (51.9)		
Combination of oral medication and insulin	10 (83.3)	2 (16.7)		
Polyhydramnios			20.0^b^	<0.001
Yes	10 (83.3)	2 (16.7)		
No	46 (24.0)	146 (76.0)		
Macrosomic baby			12.2^b^	<0.001
Yes	9 (69.2)	4(30.8)		
No	47 (24.6)	144 (75.4)		
Congenital anomaly			2.35^b^	0.183
Yes	2 (66.7)	1 (33.3)		
No	54 (26.9)	147 (3.1)		
First-degree family history of DM			5.49^c^	0.019
Yes	48 (31.8)	103 (68.2)		
No	8 (15.1)	45 (84.9)		
Physical activity			37.3^c^	<0.001
Active	4 (7.1)	52 (92.9)		
Inactive	46 (47.4)	51 (52.6)		
Minimally active	6 (11.8)	45 (88.2)		
**Diagnostic OGTT result (mmol/L)**				
Fasting blood glucose level	5.2 (0.71)	5.1 (0.61)	0.416^a^	0.678
2-h postprandial glucose level	8.8 (1.17)	8.2 (1.15)	3.502^a^	<0.001

a Independent t-test

b Fisher’s exact test

c Pearson chi-square test. Odds ratios were unadjusted (bivariate analysis).

The multivariable analysis revealed several independent predictors of postpartum glucose intolerance among the participants, as summarised in Table 4. All non-significant factors (P>0.05) in the full model were step-wise removed to create the reduced model. After refinement of the model, it remained a strong predictor, with slight decreases in the R2 and AUC values, indicating its reliability.

**Table 4:** Multivariable analysis of the factors associated with glucose intolerance among the participants (N=204).

Factor	Full model		Reduced model	
	aOR (95% CI)	P-value	aOR (95% CI)	P-value
Age (year)	1.09 (0.99, 1.21)	0.077		
**Economic status**				
B40 (R)	-		-	
M40	0.23 (0.07, 0.74)	0.013	0.22 (0.08, 0.58)	0.003
T20	0.43 (0.09, 2.16)	0.306	0.37 (0.09, 1.45)	0.152
**Employment status**				
Unemployed (R)	-		-	
Employed	2.87 (0.99, 8.33)	0.052	2.77 (1.07, 7.14)	0.035
**Parity**				
<5 (R)	-			
≥5	0.252 (0.04, 1.43)	0.120		
**BMI at booking**				
Normal (R)	-			
Underweight	3.16 (0.25, 39.27)	0.371		
Overweight	1.19 (0.35, 4.05)	0.785		
Obesity class 1	1.51 (0.39, 5.82)	0.548		
Obesity class 2	13.11 (1.48, 116.05)	0.021		
Obesity class 3	0.72 (0.02, 18.73)	0.841		
**Gestational weight gain**				
Normal (R)	-			
Low	0.69 (0.19, 2.52)	0.57		
Excessive	0.67 (0.20, 2.19)	0.507		
**GDM treatment**				
Diet control (R)	-			
Oral medication	3.91 (1.36, 11.29)	0.012	4.22 (1.74, 10.23)	0.001
Combination of oral medication and insulin	7.44 (0.46, 120.54)	0.158	14.22 (1.81, 111.39)	0.012
**Polyhydramnios**				
No (R)	-		-	
Yes	52.18 (3.30, 826.30)	0.005	31.25 (2.1, 463.91)	0.012
**Macrosomic baby**				
No (R)	-			
Yes	8.028 (1.01, 63.89)	0.049		
**Congenital anomaly**				
No (R)	-			
Yes	2.62 (2.38^e-5, 288,871.76)	0.871		
**First-degree family history of DM**				
No (R)	-			
Yes	1.25 (0.37, 4.19)	0.720		
**Physical activity**				
Inactive (R)	-		-	
Active	0.05 (0.01, 0.23)	<0.001	0.07 (0.02, 0.27)	<0.001
Minimally active	0.14 (0.04, 0.48)	0.002	0.13 (0.04, 0.42)	<0.001
2-h postprandial glucose level (mmol/L)	1.90 (1.20, 3.01)	0.006	1.82 (1.20, 2.76)	0.005
Nagelkerke R^2^	0.599		0.533	
AUC	0.900		0.877	

aOR: adjusted odds ratio, CI: confidence interval, (R): reference group, AUC: area under the curve.

The participants in the B40 economic group were at a significantly higher risk of glucose intolerance than their counterparts in the M40 group, with the odds being reduced by 78% for the latter (adjusted odds ratio [a0R]=0.22, 95% CI=0.08-0.58, P=0.003). Employment status was significant, with the employed participants being 2.77 times more likely to experience postpartum glucose intolerance than the unemployed participants.

The treatment modality for GDM during pregnancy strongly predicted the postpartum glucose outcome. The participants on oral medication had 4.22 times higher odds of having postpartum glucose intolerance (a0R=4.22, 95% CI=1.74-10.23, P=0.001), while those on a combination of oral medication and insulin exhibited 14.22 times higher odds (a0R=14.22, 95% CI= 1.81—111.39, P=0.012). The presence of polyhydramnios further increased the odds by over 31 times (a0R=31.25, 95% CI=2.10-463.91, P=0.012). Additionally, every 1-mmol/L increase in the 2-h postprandial OGTT results led to a nearly twofold increase in the odds of developing postpartum glucose intolerance (a0R=1.82, 95% CI=1.20-2.76, P=0.005).

Conversely, engaging in physical activity showed a protective effect against postpartum glucose intolerance, with the minimally active participants showing decreased odds (a0R=0.13, 95% CI=0.04—0.42, P<0.001) and the highly active participants demonstrating the lowest risk (a0R=0.07, 95% CI=0.02-0.27, P<0.001).

## Discussion

### Prevalence of glucose intolerance among postpartum women

The prevalence of abnormal glucose tolerance among postpartum women with a history of GDM in this study was 27.5%, including prediabetes affecting 23.5% and diabetes affecting 3.9%. Across Malaysia, the prevalence of postpartum glucose intolerance has ranged from 12.1% to 61.7%.^6,9,10,18^ A study conducted in Putrajaya found that the prevalence of prediabetes and diabetes among postpartum women with a history of GDM was 13.2% and 2.1%, respectively. 10 In contrast, a study in Kuala Lumpur, covering a postpartum period of 3 months to 15 years, reported a much higher prevalence at 61.7%, with 26.2% for prediabetes and 35.5% for diabetes.^9^ This variability is attributed to differences in compliance rates, assessment criteria and study settings.^6,9,10,18^ Although 75-g 2-h OGTT was consistently used across all studies as the primary diagnostic tool, the screening timelines varied significantly, ranging from 6 weeks to 15 years postpartum.^6,9,10,18^ In the context of this study, the women underwent screening at 6-12 weeks postpartum due to logistical considerations. This timeframe provided results that align more closely with studies using similar screening intervals.^6,7^

Globally, the prevalence of glucose intolerance among postpartum women varies from 13.5% to 21.4%, potentially due to differences in study populations, diagnostic criteria and timing of postpartum testing.^19-21^ Previous studies have involved diverse ethnic groups, including Caucasian, African and Korean women, which may have influenced the prevalence rates due to genetic and lifestyle factors apart from diagnostic criteria and different screening time points.^19-21^ Similarly, among studies conducted in Malaysia, some assessed glucose tolerance at 6 weeks postpartum, while others used different follow-up periods, affecting prevalence estimates.

### Sociodemographic characteristics relative to glucose intolerance among postpartum women

This study explored the factors associated with postpartum glucose intolerance. In the study population, lower economic status, employment, the use of oral medication or insulin during pregnancy, polyhydramnios, elevated 2-h postprandial glucose level and physical inactivity significantly increased the risk of developing glucose intolerance among the postpartum women.

Low socioeconomic status is an independent risk factor for developing T2DM, as individuals in this economic group often face economic limitations, have lower health literacy and experience reduced access to healthcare.^22^ According to the 2019 National Health Morbidity Survey in Malaysia, diabetes prevalence is highest among the B40 group, the unemployed and those with lower educational levels.^2^ The current data align with these findings, showing that mothers in the M40 group had lower odds of developing glucose intolerance. In this study, the employed mothers exhibited higher odds of having postpartum glucose intolerance, consistent with the findings from a study conducted in Selangor, where employment was associated with postpartum glucose intolerance.^7^ Conversely, a study in the Besut District of Terengganu found no significant association.^18^ These disparities may arise from regional differences in socioeconomic conditions and access to healthcare. This may be due to competing work responsibilities that reduce time for postpartum follow-up and limit engagement in healthy lifestyle practices, despite potential financial stability. Limited access to healthcare and time constraints may therefore contribute to this higher risk.

Although studies involving Asian and Caucasian cohorts have identified advanced maternal age as an independent predictor of future T2DM among women who had GDM, our study did not find this association.^23^ This aligns with findings from studies conducted in Besut and Belgium.^18,24^ The differences can likely be explained by the variation in age ranges among the studies. Most studies that identified a significant association involved women aged over 35 years,^18,23,24^ whereas the mean age of women in our study was 31.7 years.

The study found no significant association of race and educational level with glucose intolerance. Similar results were observed in an Iranian study.^25^ However, research in Kuala Lumpur reported a higher prevalence of diabetes among Indian women,^9^ while a study in Besut found a higher prevalence among Malay women.^18^ The disparities are likely due to ethnic and population differences. The study conducted in Besut also suggested that higher educational levels may reduce postpartum diabetes occurrence by enhancing illness understanding.^18^ Variations in cultural practices and lifestyles, which influence dietary patterns and physical activity levels, along with differences in diagnostic criteria, may explain these discrepancies.

### Physical activity and clinical characteristics relative to glucose intolerance among postpartum women

Regular physical activity is widely acknowledged to reduce the risk of developing diabetes mellitus. In this study, the women who engaged in even minimal physical activities had a reduced likelihood of developing diabetes. In general, for postpartum women, engaging in regular exercise can help control weight, improve insulin sensitivity and reduce blood sugar levels, thereby reducing the risk of diabetes. Notably, no local studies have investigated the impact of physical activity on diabetes development among postpartum women with GDM.^6,9,10,18^ Reports have suggested that an active lifestyle before pregnancy is associated with a reduced occurrence of diabetes and that low physical activity is linked to a higher risk of progression to diabetes.^30,31^

This study also found that the women who received pharmacotherapy during pregnancy for GDM were more likely to develop postpartum glucose intolerance. Insulin therapy, in particular, suggests worsening hyperglycaemia and impaired P-cell function, emphasising the need for early detection in this vulnerable group.^9^ This finding aligns with a report from Johor, which also linked insulin use with an increased risk of postpartum glucose intolerance.^6^ Additionally, a German study reported that 92.3% of women using insulin during pregnancy developed diabetes within 15 years,^26^ while a Chinese study suggested that insulin use during pregnancy raises the risk of GDM in future pregnancies.^27^

The 2-h postprandial OGTT results at diagnosis were linked to a 1.82-fold increase in the likelihood of postpartum glucose intolerance, which is slightly higher than the 1.3-fold increase observed in a Putrajaya study.^10^ The observed differences may be attributed to the ethnic diversity of the participants. Conversely, a study conducted in China indicated that a higher 2-h postprandial OGTT result at diagnosis was associated with a 2.426-fold increased risk of developing diabetes postpartum.^28^ The consistent association between postprandial glucose levels and postpartum glucose intolerance highlights the significance of this measurement, as insulin resistance is more closely linked to postprandial glucose than to fasting glucose. This is corroborated by studies from Poland^20^ and Kuala Lumpur,^9^ highlighting the importance of postprandial glucose measurements in predicting postpartum glucose intolerance. Conversely, studies from Besut and Johor did not explore this relationship.^6,18^

A significant association was identified between polyhydramnios and postpartum glucose intolerance. The occurrence of polyhydramnios is linked to poor diabetic control.^29^ However, no additional studies have directly connected polyhydramnios with the development of postpartum glucose intolerance. Due to the limited direct evidence, further research is needed to explore this relationship in greater detail.

### Strengths and limitations

Our study is among the few studies conducted in Penang, a northern region in Malaysia. It recruited women from four districts, representing both urban and suburban areas. It identified high-risk groups for glucose intolerance, emphasising the role of modifiable risk factors such as physical activity, which has not been widely explored in previous Malaysian studies. This provides important context-specific insights for local primary care practice.

Compared with similar previous studies,^4,9,10^ our study explored the role of physical activities. However, it did not extensively examine other lifestyle factors, such as dietary habits, stress levels and sleep patterns, which are known to influence the development of glucose intolerance and diabetes. The wide range of postpartum durations for physical activity assessment in this study - from 42 to 90 days - should be acknowledged as a limitation, as it can influence recovery and physical activity levels. Standardising the postpartum duration and considering the type of delivery could improve the study by ensuring a more consistent physical activity capacity among all participants.

The relatively small sample size is another limitation, which may reduce the generalisability of our findings. While the sample size was calculated to ensure adequate statistical power for the regression analysis, larger-scale and longer-term cohort studies would provide more robust prevalence estimates.

Finally, the cross-sectional study design provided a valuable overview of the health conditions and risk factors within the study population, making it a cost-effective and efficient method for generating hypotheses. However, its key limitations include the inability to establish causality, susceptibility to recall and selection bias and challenge of determining temporal relationships. Additionally, the use of convenience sampling in this study may introduce selection bias. Despite these limitations, cross-sectional studies remain an important tool in primary care research. Our findings offer valuable insights for developing targeted interventions and improving maternal health outcomes by exploring the link between these modifiable risk factors and glucose intolerance.

### Implications for future research and clinical practice

We did not explore the underlying reasons for non-compliance with postpartum glucose testing, which likely impacts the accuracy of the reported prevalence of glucose intolerance. This limitation highlights the need for further research to identify the barriers to postpartum testing among women with GDM. Understanding and addressing these barriers are crucial for ensuring accurate prevalence data and developing context-specific interventions to improve postpartum care and health outcomes in this population.

Our study highlights the critical importance of incorporating physical activity into prepregnancy care to reduce the risk of postpartum glucose intolerance. Regular exercise should be emphasised in pre-pregnancy counselling as a key preventive measure. Integrating these insights into prenatal and postpartum programmes could significantly decrease the incidence of GDM and subsequent postpartum diabetes, ultimately improving long-term health outcomes for both mothers and their children.

## Conclusion

In conclusion, the prevalence of glucose intolerance among mothers with a history of GDM in Penang was 27.5%. The multivariable analysis identified six significant factors associated with postpartum glucose intolerance, including the use of oral medication and insulin during pregnancy and physical inactivity. These factors can be utilised to identify mothers at a higher risk of postpartum diabetes and prediabetes during the antenatal period. Implementing targeted interventions, such as tailored physical activity programmes and comprehensive postpartum education, is essential to mitigate the risk of postpartum glucose intolerance in this population.

## Data sharing statement

The data generated and analysed during the study are available from the corresponding author upon reasonable request.

**How does this paper make a difference in general practice?**

- This paper emphasises the importance of adherence to 6-week postpartum glucose testing.
- It encourages primary care practitioners to prioritise postpartum screenings for early detection of prediabetes or diabetes.
- The study fills a research gap by being one of the few studies in Malaysia that incorporated lifestyle risk factors such as physical activities in association with postpartum glucose intolerance.
